# Age-specific differences in gait domains and global cognitive function in older women: gait characteristics based on gait speed modification

**DOI:** 10.7717/peerj.8820

**Published:** 2020-03-16

**Authors:** Byungjoo Noh, Changhong Youm, Myeounggon Lee, Hwayoung Park

**Affiliations:** 1 Department of Health Care and Science, College of Health Sciences, Dong-A University, Busan, Republic of Korea; 2 Biomechanics Laboratory, College of Health Sciences, Dong-A University, Busan, Republic of Korea

**Keywords:** Gait, Older women, Global cognitive function, Inertial measurement unit, Walking, Wearable sensor

## Abstract

**Background:**

Several studies have reported the association between gait and global cognitive function; however, there is no study explaining the age-specific gait characteristics of older women and association between those characteristics and global cognitive function by age-specific differences and gait speed modification. The aim of this study was to examine age-specific differences in gait characteristics and global cognitive function in older women as well as identify gait domains strongly associated with global cognitive function in older women based on gait speed modification.

**Methods:**

One hundred sixty-four female participants aged 65–85 years were examined. Participants were assessed for global cognitive function through the mini-mental state examination. They also performed three trials of the overground walking test along a straight 20 m walkway. Inertial measurement unit sensors with shoe-type data loggers on both the left and right outsoles were used to measure gait characteristics.

**Results:**

The pace at all speeds and the variability and phase at faster speeds were altered in women aged >75 years (all pace domain parameters, *p* < 0.05); variability and phase highly depended on age (all *p* < 0.05). Variability at slower speeds (β = −0.568 and *p* = 0.006) and the phase at the preferred (β = −0.471 and *p* = 0.005) and faster speeds (β = −0.494 and *p* = 0.005) were associated with global cognitive function in women aged >75 years.

**Discussion:**

The variability and phase domains at faster speeds were considered to identify gait changes that accompany aging. In addition, the decreases in global cognitive function are associated with increased variability and phase domains caused by changes in gait speed in older women.

**Conclusion:**

Our results are considered useful for understanding age-related gait characteristics with global cognitive function in old women.

## Introduction

Gait is considered to have a cognitive component because of modulation during the descending drive from the brainstem to the spinal cord that involves a higher level of cognitive functioning (Hausdorff et al., 2005). Age-related impairments of gait are linked to structural and functional brain changes, commonly occurring with aging (Holtzer, Wang & Verghese, 2012; Holtzer et al., 2014). Consequently, gait abnormality is associated with the risks of dementia, which is one of the most common cognitive disease in older adults (Verghese et al., 2007). Furthermore, women are more vulnerable to cognitive impairment than men (Azad, Al Bugami & Loy-English, 2007; Walston & Fried, 1999), which results in a greater predisposition toward age-related gait changes and health problems, thus leading to a lower quality of life.

Several studies investigated the associations between gait domains and decrements in global cognitive function in older adults. Impairments of the motor system such as gait abnormality occur during the early stages of dementia (Albers et al., 2015). The pace of gait (especially, slow walking) is a predicting marker for identifying cognitive decline or risks of dementia (Aggarwal et al., 2006; Mielke et al., 2013). Moreover, high gait variability of step length and stance time is associated with executive function (Hausdorff et al., 2005; Verlinden et al., 2014) as well as mild cognitive impairment (Beauchet et al., 2013; Verghese et al., 2008). Furthermore, the phase domain is associated with memory (Verlinden et al., 2014). Therefore, various gait domains should be considered to identify gait-domain-related declines in cognitive functions.

Although several studies regarding gait and global cognitive function exist, the results are inconsistent and the relationship between gait and global cognitive function remains unclear in terms of which domains or variables are related (Morris et al., 2016). Furthermore, there are limited studies that have examined age-specific differences in gait characteristics and the declining cognitive functions using wearable inertial measurement units, which can measure a more continuative states for longer durations in the real-world at relatively low cost than a motion capture system. Thus, research on age-related changes in walking abilities and global cognitive functions, and possible predictors of cognitive function decline in older women is necessary for gait assessment. Additionally, research in this regard should consider more sensitive indicators of gait abnormalities such as variability and phase domains rather than pace (walking speed), which may lack specificity.

Therefore, the primary aim of this study was to examine age-specific differences in gait characteristics and global cognitive function in older women according to gait speed modification, and it must investigate the gait domains that reflect age-related gait differences. The secondary aim was to investigate different parameters associated with the gait domains and global cognitive function according to gait speed modification in older women. We hypothesized that older women advancing in age would show altered gait domains and mini-mental state examination (MMSE) scores according to gait speed modification. We also suspected that decrements in the MMSE scores could be further associated with alterations in gait domains according to gait speed modification, which involves pace, rhythm, variability, phase and asymmetry in older women.

## Materials and Methods

### Participants

**Figure 1:**
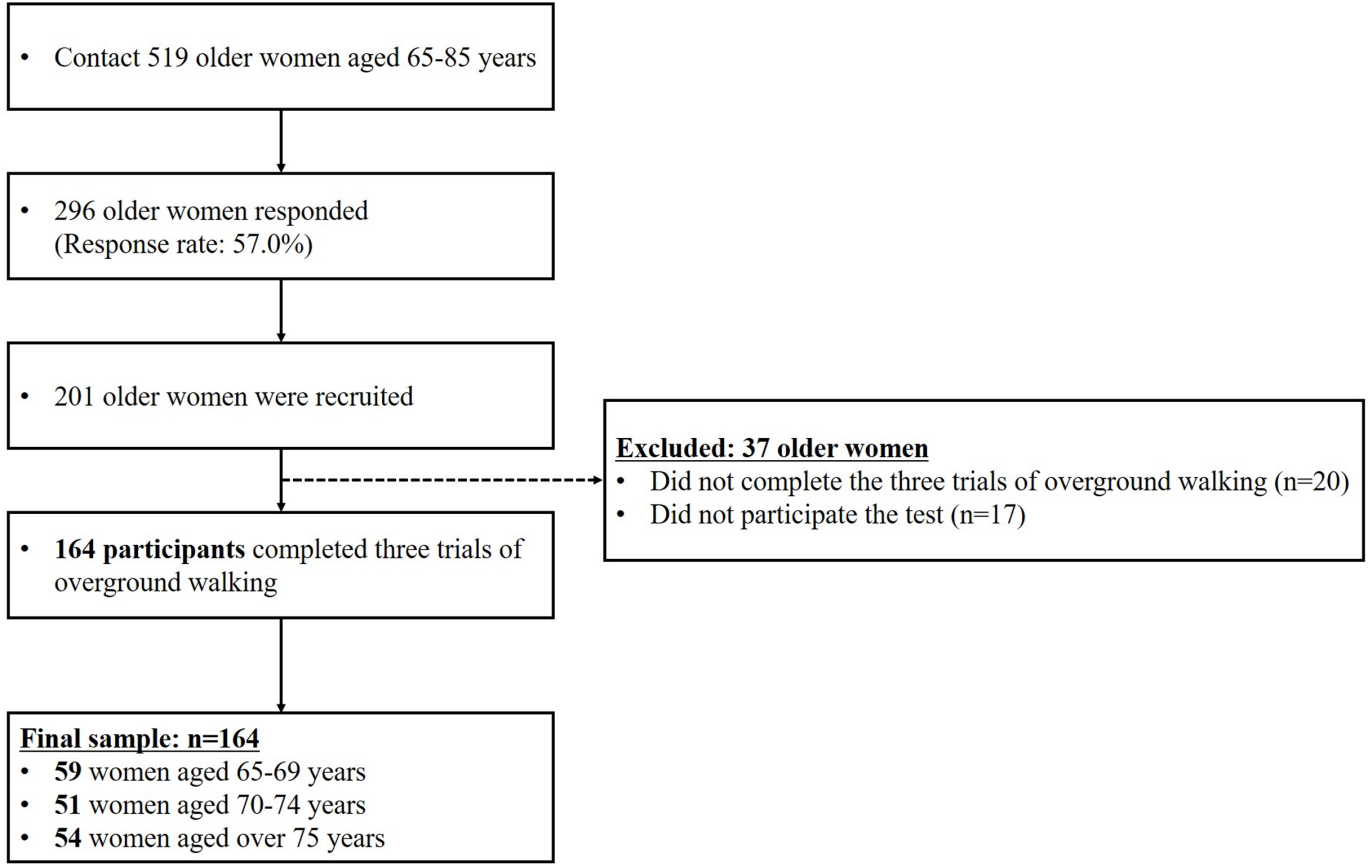
Flow diagram explaining criteria for participant selection.

Participants for this study were recruited as part of a community-wide survey in Busan metropolitan city in 2018–2019. We contacted to 519 women aged 65 years and over living in the community, with 296 participants responding (response rate: 57.0%). Participants unable to walk without any support and with a history of severe orthopedic problems or neurosurgical and neurophysiological problems in the preceding six months were excluded. Ultimately, 164 community-dwelling cognitively normal older women aged 65 years and over participated in the overground walking test (Fig. 1; Table 1). All the participants read and signed an informed consent form that was approved by the Institutional Review Board of Dong-A University (IRB number: 2-104709-AB-N-01-201808-HR-023-02).

**Table 1:** Demographic characteristics.

	Aged 65–69 (*n* = 59)	Aged 70–74 (*n* = 51)	Aged over 75 (*n* = 54)	All participants (*n* = 164)	*p*-Value	Post-hoc
Age (years)	67.3 ± 1.3	72.0 ± 1.3	77.9 ± 2.3	72.3 ± 4.7	**<0.001**	a, b, c
Height (cm)	152.4 ± 5.1	152.4 ± 5.3	151.7 ± 4.6	152.2 ± 5.0	0.709	
Body weight (kg)	58.2 ± 6.8	59.0 ± 7.4	60.1 ± 7.9	59.1 ± 7.4	0.388	
BMI (kg/m^2^)	25.2 ± 3.0	25.4 ± 2.9	26.1 ± 3.2	25.6 ± 3.1	0.264	
Body fat (%)	35.4 ± 5.5	36.8 ± 4.7	37.1 ± 5.0	36.4 ± 5.1	0.182	
Total PA (MET-min/week)	2,426.3 ± 2,321.8	2,046.5 ± 1,354.8	949.8 ± 889.5	1,822.0 ± 1,771.6	**<0.001**	b, c
Education (years)	8.3 ± 2.3	7.9 ± 2.2	7.3 ± 2.2	7.8 ± 2.2	0.076	
ICC (slower)	0.77	0.87	0.88	0.85	**–**	
ICC (faster)	0.99	0.99	0.99	0.99	–	

**Note:**

Mean ± SD, mean and standard deviation; BMI, body mass index; PA: physical activity; METs, metabolic equivalents; ICC, intraclass correlation coefficient, boldface denotes a significant difference between age groups, Post-hoc: a, aged 65–69 vs. aged 70–74; b, aged 70–74 vs. aged over 75; c, aged 65–69 vs. aged over 75; significant difference, *p* < 0.0167.

### Instrumentation

**Figure 2:**
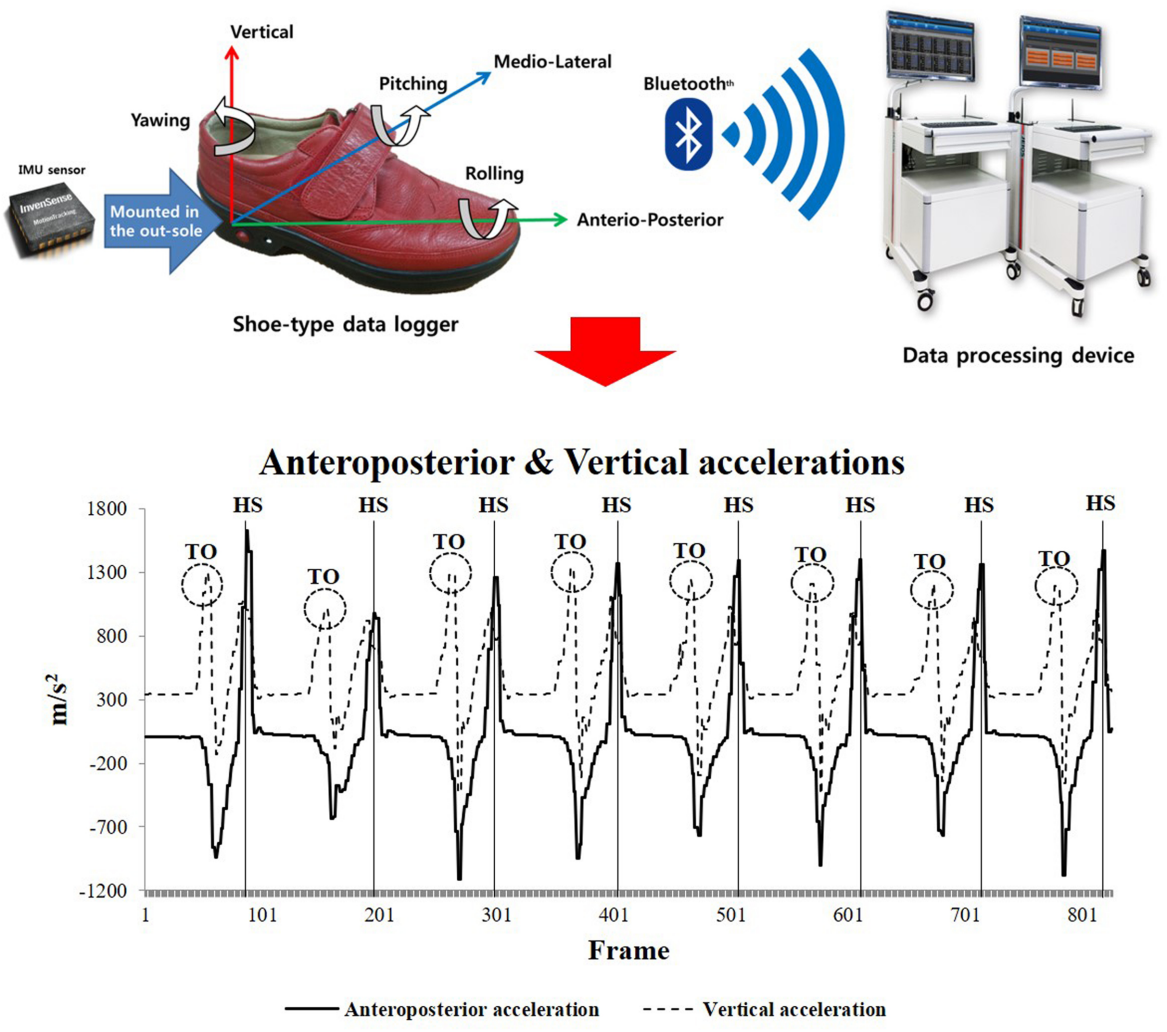
Shoe-type IMU system and detection of gait events (HS represents heel strike, and TO represents toe off) (Lee et al., 2018).

We used a shoe-type gait analysis system based on an IMU sensor (DynaStab™, JEIOS, Busan, South Korea) composed of shoe-type data loggers (Smart Balance SB-1; JEIOS, South Korea) with a data acquisition system (Lee et al., 2018). The shoe-type data logger included IMU sensors on both the left and right outsoles (IMU-3000TM; InvenSense, San Jose, CA, USA); these were used to measure triaxial acceleration (up to ±6 g) and triaxial angular velocities (up to ±500° s^−1^) along three orthogonal axes (Kim et al., 2016; Lee et al., 2018). The data were transmitted wirelessly to a data acquisition system via Bluetooth, and multiple shoe sizes were available to fit all participants. The local coordinate system of the IMU sensors was established in the anteroposterior, mediolateral and vertical directions (Fig. 2).

### Test procedures

Prior to the overground walking test, biometric data, including the body height, weight, and body fat percentage, of all the participants were recorded. All participants completed a questionnaire to assess their physical activity (PA) levels. Habitual PA levels were assessed by a self-reported questionnaire (international PA questionnaire-short form, IPAQ-SF). All participants performed a warm-up protocol comprising a stretching program. They performed the overground walking test wearing the shoe-type embedded IMU sensors.

### Global cognitive function assessments

The MMSE was used to assess global cognitive function (Folstein, Folstein & McHugh, 1975); this is the most common screening tool for dementia and for assessing global cognitive function. All participants completed this questionnaire and we assessed their responses in terms of their global cognition. It is a 30-point questionnaire that includes tests pertaining to orientation, attention, memory, language and visual-spatial skills.

### Gait performance measures

All participants completed three trials of the overground walking test along a straight 20 m walkway (approximately 40 steps) at slower (80% of usual), preferred (usual walking), and faster (120% of usual) speeds. The preferred speed refers to the comfortable and stable walking speed of the participant without any support during overground walking. The walking speeds were defined using a metronome (beats/min). Each speed was calculated relative to the preferred speed, that is, 20% slower or faster than the preferred speed (Chung & Wang, 2010). The participant was asked to walk at the preferred speed to measure cadence using a metronome before each trial. An experimental operator mentioned the walking speeds to the participants before each trial, and the participants were required to perform the overground walking test at the speed that was as close as possible to the target walking speed paced by a metronome. The intraclass correlation coefficient (ICC) was calculated to distinguish between the real and calculated speed consistency at slower and faster speeds. They were also given verbal or visual instructions to perform overground walking and practiced walking under all speed conditions with the metronome for approximately 10 min during a familiarization session.

### Data analysis

The overground walking data were collected at 100 Hz. The shoe-type IMU system data were filtered using a second-order Butterworth low-pass filter with a cutoff frequency of 10 Hz (Kim et al., 2016; Lee et al., 2018). The two initial acceleration steps and final deceleration steps at the end of the test were excluded as we intended to analyze only the consecutive steps in the steady-state condition (Fig. 2). Gait events were identified as heel strikes that occurred when the linear acceleration along the anteroposterior axis reached its maximum value. Subsequently, toe-offs occurred when the linear acceleration along the vertical axis reached its maximum value during a gait cycle (Kim et al., 2016; Lee et al., 2018).

The spatiotemporal parameters, such as the walking speed, cadence, stride length, step length, stride time, step time, single support phase, double support phase and stance phase, were calculated. The walking speed was calculated the average speed as time for the 20 m walk from an IMU sensor (time/distance); This was converted to meter per second. The normalized spatiotemporal parameters were divided by the height of each participant. In addition, the coefficient of variance (CV (standard deviation/mean) × 100) values for all spatiotemporal parameters were calculated. The gait asymmetry (GA) was measured using the differences between the left and right movements during walking (Plotnik, Giladi & Hausdorff, 2007). These parameters can be categorized into the following gait domains: pace (walking speed, stride length, step length and normalized variables), rhythm (cadence, stride and step time), phase (single support, double support and stance phase), variability (CV for spatiotemporal parameters) and asymmetry (GA) (Lord et al., 2013).

### Statistical analysis

All statistical analyses were performed using SPSS for Windows (version 25.0; IBM Corp., Armonk, NY, USA). The Shapiro–Wilk test was used to determine whether the data had a normal distribution. Before additional analysis, Z-normalization (value–mean/standard deviation) of all variables was performed. Especially, Z-normalization was performed to assess the global cognitive function because the MMSE score is not used as a continuous variable. Subsequently, one-way analysis of variance with the Bonferroni post-hoc test was used to compare the age-specific differences in the MMSE scores (MMSE cutoff < 24) and spatiotemporal parameters under different three walking speeds with the statistical significance levels set at 0.0167 (0.05/3). Multinomial logistic regression was conducted to determine the age classifiers for all participants aged 65–69 years under each walking speed that contained all confounders. Furthermore, stepwise multivariable linear regression analysis was performed to identify the independent variables and explain the significance of the dependent variables (MMSE scores). The covariates were the age, education, % body fat and PA. Previous studies reported that excessive body fat in adulthood is prospectively associated with increased risks of cognitive dysfunction and dementia in later life (Anstey et al., 2011; Loef & Walach, 2013). The level of statistical significance was set at 0.05.

## Results

### Comparisons among the age-specific groups in terms of the MMSE scores and spatiotemporal parameters

Table 2 lists only statistically significant results for the MMSE scores and gait-related variables at three different speeds for the age-specific groups that comprised cognitively normal participants. The MMSE score shows that a significant part of the subjects score <24, which defines a degree of dementia. The MMSE scores were significantly lower for women aged >75 years compared to those aged 65–69 years (*p* = 0.008). Most variables indicated significant differences between the age-specific groups, namely 65–69 years and >75 years regarding the pace (stride length, step length and normalized variables at all speeds as well as the walking speed and normalized walking speed at the preferred and faster speeds), phases (double support phase and stance phase at faster walking speed), and variability (CV of stance phase at faster walking speeds) (all *p* < 0.05). In addition, the pace (stride length, step length and normalized variables at all speeds as well as the walking speed and normalized walking speed at the preferred speed) showed significant differences between the groups aged 70–74 years and >75 years (all *p* < 0.05). The double support phase and stance phase at faster walking speeds showed significant differences between groups aged 65–69 years and 70–74 years (all *p* < 0.05).

**Table 2:** Comparison among age-specific groups in terms of MMSE scores and spatiotemporal parameters.

	Aged 65–69	Aged 70–74	Aged over 75	Effect size (η^2^)	Post-hoc	
**MMSE score**	27.02 ± 2.19	26.00 ± 2.17	25.48 ± 3.45	0.06	c	
**Slower speed**						
Step length (m)	0.59 ± 0.06	0.58 ± 0.06	0.55 ± 0.07	0.09	b, c	
Stride length (m)	1.18 ± 0.12	1.17 ± 0.13	1.09 ± 0.14	0.09	b, c	
N step length (m/height)	0.39 ± 0.04	0.38 ± 0.04	0.36 ± 0.05	0.08	b, c	
N stride length (m/height)	0.77 ± 0.08	0.77 ± 0.08	0.72 ± 0.09	0.08	b, c	
**Preferred speed**						
Walking speed (m/s)	1.26 ± 0.16	1.23 ± 0.21	1.13 ± 0.17	0.08	b, c	
Step length (m)	0.64 ± 0.06	0.63 ± 0.07	0.59 ± 0.07	0.11	b, c	
Stride length (m)	1.28 ± 0.12	1.25 ± 0.15	1.17 ± 0.14	0.11	b, c	
N walking speed (m/s/height)	0.82 ± 0.11	0.80 ± 0.13	0.75 ± 0.11	0.07	b, c	
N step length (m/height)	0.42 ± 0.04	0.41 ± 0.05	0.39 ± 0.05	0.10	b, c	
N stride length (m/height)	0.84 ± 0.08	0.82 ± 0.09	0.77 ± 0.09	0.10	b, c	
**Faster speed**						
Walking speed (m/s)	1.61 ± 0.19	1.52 ± 0.19	1.45 ± 0.22	0.09	c	
Step length (m)	0.71 ± 0.06	0.70 ± 0.06	0.66 ± 0.08	0.10	b, c	
Stride length (m)	1.42 ± 0.13	1.39 ± 0.12	1.32 ± 0.15	0.10	b, c	
N walking speed (m/s/height)	1.05 ± 0.13	1.00 ± 0.12	0.96 ± 0.15	0.09	c	
N step length (m/height)	0.47 ± 0.04	0.46 ± 0.04	0.43 ± 0.05	0.09	b, c	
N stride length (m/height)	0.93 ± 0.08	0.91 ± 0.08	0.87 ± 0.10	0.09	b, c	
Double support phase (%)	16.07 ± 2.64	17.39 ± 2.65	17.35 ± 2.72	0.05	a, c	
Stance phase (%)	57.87 ± 1.36	58.58 ± 1.50	58.62 ± 1.47	0.06	a, c	
CV of stance phase (%)	2.04 ± 1.01	2.11 ± 0.64	2.48 ± 1.01	0.04	c	

**Note:**

Mean ± SD, means and standard deviations; MMSE, mini-mental state examination; N, normalized; CV, coefficient of variance; η^2^, sum of squares between groups and total of squares. Post-hoc: a, aged 65–69 vs. aged 70–74; b, aged 70–74 vs. aged over 75; c, aged 65–69 vs. aged over 75; significant difference, *p* < 0.0167.

### Multinomial logistic regression results

Table 3 summarizes only statistically significant results from the multinomial logistic regression for all participants. In the multinomial logistic regression models adjusted for confounders, gait-related variables at each walking speed, namely the walking speed, cadence, stride length, step length, stride time, step time, single support phase, double support phase, stance phase, CVs of the variables (stride length, step length, single support phase, double support phase and stance phase) and GA were considered.

**Table 3:** Multinomial logistic regression model for age groups under three different speeds.

Variable	Aged 65–69 years	Aged 70–74 years		Aged over 75 years	
	Odds ratio	Odds ratio	95% CI	Odds ratio	95% CI
**Slower speed**					
GA	1.0	1.05	[0.70–1.58]	1.51*	[1.03–2.20]
**Preferred speed**					
Double support phase	1.0	1.31	[0.89–1.92]	1.53*	[1.03–2.25]
Stance phase	1.0	1.31	[0.89–1.94]	1.58*	[1.07–2.23]
**Faster speed**					
Step time	1.0	1.34	[0.89–2.03]	1.64*	[1.09–2.46]
Stride time	1.0	1.57*	[1.06–2.35]	1.47	[0.99–2.17]
Double support phase	1.0	1.69*	[1.12–2.54]	1.66*	[1.11–2.48]
Stance phase	1.0	1.70*	[1.13–2.56]	1.74*	[1.16–2.61]
CV of step length	1.0	1.00	[0.64–1.57]	1.55*	[1.03–2.31]
CV of single support phase	1.0	1.42	[0.92–2.21]	1.55*	[1.00–2.39]
CV of stance phase	1.0	1.11	[0.71–1.72]	1.63*	[1.08–2.46]

**Notes:**

* Significant difference, *p* < 0.05.

Reference: aged 65–69 years; CI, confidence interval; GA, gait asymmetry; CV, coefficient of variance.

When variables associated with slower walking speeds were added to the adjusted model, GA in women aged >75 years was associated with age based on the results obtained for women aged 65–69 years (OR = 1.51 and *p* < 0.05). As for the variables associated with the preferred walking speeds, the double support phase (OR = 1.53 and *p* < 0.05) and stance phase (OR = 1.58 and *p* < 0.05) in women aged >75 years were associated with age based on the results obtained for women aged 65–69 years. At faster walking speeds, the stride time (OR = 1.57 and *p* < 0.05), double support phase (OR = 1.69 and *p* < 0.05), and stance phase (OR = 1.70 and *p* < 0.05) for women aged 70–74 years were associated with age based on the results for women aged 65–69 years, which were added to the adjusted model. In addition, the step time (OR = 1.64 and *p* < 0.05), double support phase (OR = 1.66 and *p* < 0.05), stance phase (OR = 1.74 and *p* < 0.05), and the CVs of the step length (OR = 1.55 and *p* < 0.05), single support phase (OR = 1.55 and *p* < 0.05), and stance phase (OR = 1.63 and *p* < 0.05) in women aged >75 years were associated with age based on the results for women aged 65–69 years when variables associated with faster walking speeds were added to the adjusted model.

### Results of stepwise multivariable linear regression analysis

Table 4 lists only statistically significant results for the associations between gait parameters based on the MMSE scores for older women at three different speeds. After adjustments to the confounders, the MMSE scores for all participants were significantly associated with the double support phase (slower, β = −0.173 and *p* = 0.024) and stance phase (preferred, β = −0.242 and *p* = 0.002; faster, β = −0.245 and *p* = 0.002). For women aged 70–74 years, cadence (β = −0.280 and *p* = 0.018) was significantly associated with the MMSE score at faster walking speeds. In addition, the CVs of the stride length (slower, β = −0.568 and *p* = 0.006) and stance phase (preferred, β = −0.471 and *p* = 0.005; faster, β = −0.494 and *p* = 0.005) were significantly associated with the MMSE score in women aged >75 years.

**Table 4:** Association of gait parameters with MMSE scoresunder three different speeds in old women.

Variable	MMSE score		
	β (SE)	*t*	*p*-Value
**All participants**			
Double support phase (slower)	−0.173 (0,076)	2.285	**0.024**
Stance phase (preferred)	−0.242 (0.077)	3.144	**0.002**
Stance phase (faster)	−0.245 (0.077)	3.199	**0.002**
**Aged 70–74 years**			
Cadence (faster)	−0.280 (0.114)	2.449	**0.018**
**Aged over 75 years**			
CV of stride length (slower)	−0.568 (0.199)	2.862	**0.006**
Stance phase (preferred)	−0.471 (0.159)	2.967	**0.005**
Stance phase (faster)	−0.494 (0.166)	2.972	**0.005**

**Note:**

Model adjusted for age, education, % body fat, and physical activity. MMSE, mini-mental state examination; SE, standard error; CV, coefficient of variance; boldface denotes a significant difference.

## Discussion

This study analyzed the age-specific differences in gait domains and the association between the gait domains and global cognitive functions at three different walking speeds. The main findings of this study are as follows: (1) The pace domains at all speeds and variability and phase domains at faster speeds were altered in women aged >75 years. (2) The variability and phase domain were the variables that highly depended on age. (3) The variability domain at slower speeds and phase domain at the preferred and faster speed were associated with global cognitive function in women aged >75 years. These findings are discussed in detail below.

### Age-specific differences in gait domains according to gait speed modification in older women

At the preferred speed, our findings of step length and cadence yielded similar results compared with a previous study in women aged 60–69 years (Senden et al., 2009) (present vs. reference, step length: 0.64 ± 0.06 m vs. 0.66 ± 0.07 m; cadence: 117.39 ± 9.19 st/min vs. 116.53 ± 5.60 st/min). These findings imply that our findings were as good as those found in the other study (Senden et al., 2009). These results help validate our findings, which is necessary for clinical use.

Numerous studies have obtained findings similar to our study (Van Kan et al., 2009; Cesari et al., 2005; Studenski et al., 2011). Our study indicated that there were age-related decrements in the pace domain at three different walking speeds in older women with lower PA and education levels. Interestingly, phase domain parameters increased until people reached 70 years and then became steady whereas the pace domain parameters decreased steadily with age. This state may occur because of the motor dysfunction associated with age-related reduction in muscle power (Senefeld, Yoon & Hunter, 2017; Thom et al., 2007; Thompson et al., 2013), requiring economical walking with minimal energy expenditure as a compensative strategy. However, this state was observed at faster walking speeds only in our study. Our logistic analysis showed age-specific changes in the phase domain in women aged 70–74 years; moreover, the phase and variability domains in women aged >75 years were highly dependent on aging. Therefore, these two domains may reflect the aging effects better than other gait domains. Thus, analyzing the effects of aging on gait, it is required to identify age-specific changes with more detail age classifications including people aged >85 years.

### Age-specific association between gait domains and global cognitive function according to gait speed modification

Previous studies reported that the variability domain parameter is most strongly associated with cognitive function (Hausdorff et al., 2005; Martin et al., 2013; Van Iersel et al., 2008; Verlinden et al., 2014). Likewise, our study also showed that the higher CV values of the stride length was associated with the lower MMSE scores at slower walking speeds in women aged >75 years. It may be related to the executive dysfunction of cortical sensorimotor control (Beauchet et al., 2009, 2013, 2014) in response to a reduced hippocampal volume and impaired function in older adults (Annweiler et al., 2012). The executive function is related to initiating and modulating the gait performance (Senefeld, Yoon & Hunter, 2017). Therefore, high gait variability could be attributed to the stride-to-stride fluctuations during walking to generate force using muscle with the partial summation of overlapping twitches due to executive dysfunction during modulation in slow walking. Furthermore, our ICC results presented relatively lower value at slower speed (ICC, 0.85) than faster speed (ICC, 0.99). Therefore, it is difficult to adjust the speed when walking at slow speeds for older women with declining cognitive function. Thus, the variability domain at slower walking speeds can be a more sensitive predictor for identifying future cognitive decline than other gait domains at relatively fast walking speeds (Beauchet et al., 2013; Verghese et al., 2008).

Our findings are similar to previous studies that analyzed the association between a longer stance phase and lower MMSE score from the preferred to the faster walking speed in women aged >75 years. Very few studies have shown that phase domain parameters showed the strongest relationship with cognitive function (Taniguchi et al., 2019) involving memory (Verlinden et al., 2014). In addition, a longer stance phase may result from the force to length relationship. The optimal propulsion force during walking could be achieved at optimal step lengths; however, shorter step lengths lead to decreased walking speeds owing to a longer stance phase. This gait pattern as a compensative strategy (increased stance phase with slow walking speed) increases dynamic instability and could lead to falls (Aboutorabi et al., 2016). This pattern appears to result from the deterioration of locomotion as cognitive function declines, to which women may be more vulnerable than men. This finding is supported by the fact that overlapped areas of the basal ganglia and cerebellum are major subcortical structures that cooperate to control the cognitive and motor functions as well as walking (Holtzer et al., 2014; Kikkert et al., 2016). Furthermore, Iosa et al. (2013) and Serrao et al. (2017) emphasized that the golden ratio (60–62% of stance phase vs. 40–38% of swing phase) between the duration of the stance and swing phases of a gait cycle, which is the gait harmony. In particular, the loss of gait harmony could be disrupted in response to the damage in the cerebellum or basal ganglia (Serrao et al., 2017). In addition, gait stability is highly dependent on the ratio between the duration of the stance and swing phases because it could alter the gait variability. Thus, the phase domain should also be considered meaningful to the understanding cognitive function decline in old women.

In addition, Darweesh et al. (2019) in their Rotterdam Study showed a tendency for the association between the phase domain and incident dementia. It may be because of the relatively fewer steps considered in their gait assessment using GAITRite™ compared to our study. Thus, a longer walking time and walkway distance are required to identify the relationship between gait and cognitive function decline.

This study had some limitations. First, all participants performed the walking at a self-controlled speed, which may have artificially changed gait parameters. However, we confirmed the ICC results which were calculated to distinguish between the real and calculated speed consistency at slower and faster speeds (*r* > 0.77). Second, our study used the MMSE scores, which have a relatively lower sensitivity (Trzepacz et al., 2015). However, the MMSE score is a widely used screening tool for dementia and global cognitive function as well as clinical evaluations. Further research is warranted to address these issues. Third, cognitively normal participants were included into this study. Despite assuming this status, we speculate that some participants could have a diagnostic level of dementia. Fourth, this study did not entirely consider the recommendations to improve the validity of research using the MMSE (Monroe & Carter, 2012); the recommendations should be considered to improve the validity of a future study using the MMSE.

## Conclusions

The findings of this study suggest that the variability and phase domains at faster speeds should be considered to identify gait changes that accompany aging. Additionally, decrements in global cognitive function are associated with increased variability and phase domains according to gait speed modification in older women. Therefore, our results are considered meaningful to the understanding of the age-related decline in gait performance with global cognitive function according to gait speed modification in old women.

Further study using instruments to assess cognitive function and longitudinal studies are needed to determine associations between not only gait domains and global cognitive functions, but also health-related physical fitness domains and global cognitive functions in older adults.

## Supplemental Information

10.7717/peerj.8820/supp-1 Supplemental Information 1 Raw data.

## Additional Information and Declarations

### Data Availability

The following information was supplied regarding data availability: The raw measurements are available as a Supplemental File.
